# Peas Out of the Pod

**DOI:** 10.5826/dpc.1102a02

**Published:** 2021-03-08

**Authors:** Theocharis N. Kirtsios, Zoe Apalla, Aimilios Lallas

**Affiliations:** 1 Second Department of Dermatology, Aristotle University, Thessaloniki, Greece; 2 First Department of Dermatology, Aristotle University, Thessaloniki, Greece

**Keywords:** melanoma in situ, dermoscopy, diagnosis congenital nevus, acral nevus, acral melanoma

## Case Presentation

**Figure 1:**
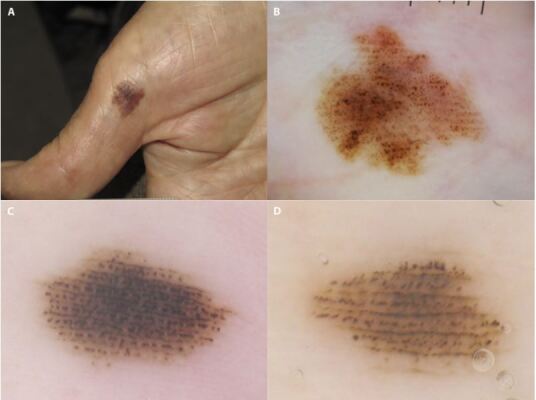
(A) A brown macule noticed on the palm of a 43-year-old woman. (B) Dermoscopy (captured with polarized dermoscopy, magnification ×10) revealed pigmentation occupying mainly the ridges and brown dots scattered all over the lesion. They were heterogeneous in size and randomly distributed, not restricted to the eccrine duct openings. The lesion was excised with the suspicion of melanoma and histopathologically diagnosed as melanoma in situ. (C, D) Examples of acral nevi, from different patients, displaying a “peas in a pod” pattern, with the brown dots distributed in parallel lines and equal distance between each other (polarized dermoscopy, magnification ×10).

A 43-year old woman presented to our department for a total-body mole check. On clinical examination, a pigmented macule was noted on her right palm (Figure 1A). The patient reported that the lesion appeared approximately 3 years earlier. Based on dermoscopic examination (Figure 1B), the lesion was assessed as suspicious for melanoma and an excision was performed. The histopathological diagnosis was melanoma in situ.

## Teaching Point

The anatomical features of acral skin produce unique and distinctive dermoscopic patterns when it comes to discriminating between acral nevi and melanoma. The diagnosis is guided by inspection of the furrows and ridges. Nevi usually display a parallel furrow pattern, consisting of parallel brown lines occupying the furrows, while the ridges are not pigmented [1]. A subtype of this pattern includes brown dots in the eccrine ducts located on the ridges (“peas in the pod”). No other distribution of brown dots is expected in a nevus [2]. When “the peas are out of the pod,” the suspicion of melanoma should be raised, as in our case.
